# Sustainable Reuse and Recycling of Spent Li‐Ion batteries from Electric Vehicles: Chemical, Environmental, and Economical Perspectives

**DOI:** 10.1002/gch2.202200212

**Published:** 2023-01-26

**Authors:** Kanit Hantanasirisakul, Montree Sawangphruk

**Affiliations:** ^1^ Centre of Excellence for Energy Storage Technology (CEST) Department of Chemical and Biomolecular Engineering School of Energy Science and Engineering Vidyasirimedhi Institute of Science and Technology Wangchan Valley Rayong 21210 Thailand

**Keywords:** battery second use, circular economy, direct recycling, hydrometallurgy, lithium‐ion batteries, pyrometallurgy, recycle

## Abstract

The rapidly increasing adoption of electric vehicles (EVs) worldwide is causing high demand for production of lithium‐ion batteries (LIBs). Tremendous efforts have been made to develop different components of LIBs in addition to design of battery pack architectures as well as manufacturing processes to make better batteries with affordable prices. Nonetheless, sustainable use of LIBs relies on the availability and cost of rare metals, which are naturally concentrated in a few countries. In addition, toxic electrolytes used in LIBs pose concerns on environmental impacts if LIBs are not handled properly after decommissioned from EVs. Therefore, it is paramount to realize effective utilization of spent LIBs, where their remaining capacities can be reused in less demanding applications. Finally, electrode materials and other valuable components of LIBs can be recovered via recycling, completing their circular life cycle. In this review, available options of LIBs after their retirement from EV applications, including battery second use, repair of electrode materials by direct regeneration, and material recovery by hydrometallurgical or pyrometallurgical processes are discussed. Throughout the review, the discussion is based around current available technologies, their environmental impacts, and economic feasibility as well as provided examples of pilot and industrial scale adoption of the processes.

## Graphical abstract

The rapidly increasing adoption of electric vehicles (EVs) worldwide is causing high demand for lithium‐ion batteries (LIBs), which inevitably leads to tremendous amount of spent and end‐of‐life LIBs in need of proper management to utilize their remaining capacity and recover valuable components, completing their circular life cycle. This review discusses currently available technologies, their environmental impacts, and economic feasibility of reuse and recycling processes of LIBs.

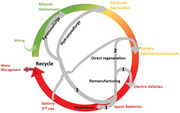

## 1 Introduction

Accelerated by the uncertainty in fossil fuel supply chain and energy price fluctuation due to global geopolitical issues as well as the pressing concerns over global warming crisis, the demand for electric vehicles (EVs) has been sharply rising in recent years. It has been projected that, globally, more than 220 million EVs will be on the road by 2030,^[^ ^1^ ^]^ which corresponds to nearly $830 billion in market value.^[^ ^2^ ^]^ Regardless of the EV type (battery‐EV, plug‐in hybrid EV, or fuel cell EV), lithium‐ion battery (LIB) is a crucial part that drives the cost of the EVs both in terms of first‐time purchase and maintenance. The increase in EV manufacturing directly means the increase in LIB production, where the annual LIB production was projected to exceed 1 million tons by 2025.^[^ ^3^ ^]^ This inevitably leads to enormous amount of off‐spec and spent LIBs that will flood the reuse and recycle markets soon. Given an average lifetime of 4000 charges or 120 000 km (≈8–10 years) before a LIB pack loses ≈20% of its original capacity which is considered unfit for the EV application, the amount of spent EV batteries that will need to be handled could reach 1.7 million tons by 2035.^[^ ^4^ ^]^ Currently, the recycling market of LIBs is estimated to be ≈$1700 million and is expected to drastically increase in response to the increase of the EV market value.^[^ ^5^ ^]^ Additionally, LIBs consist of toxic and flammable electrolyte that could severely damage the environment and pose serious risk to human health if not handled properly. Therefore, sustainable processes to reuse, repurpose, and recycle off‐spec and spent LIBs are beneficial both environmentally and economically.

A LIB cell consists of a negative electrode (anode) and a positive electrode (cathode) coated on metallic current collectors that are separated by polymeric separator soaked in an organic electrolyte. On the negative side, the anode is usually made of graphite mixed with polyvinylidene fluoride (PVDF) or styrene butadiene or carboxymethylcellulose binder coated on copper (Cu) current collector. Some advanced anode materials may contain silicon (Si) or lithium metal.^[^ ^6^ ^]^ The type of cathode materials currently being used divides LIBs into five groups, that is, lithium cobalt oxide (LCO), lithium manganese oxide (LMO), lithium nickel cobalt aluminum oxide, lithium iron phosphate (LFP), and lithium nickel manganese cobalt oxide (NMC) with a combination of the latter two types occupies ≈60% of the market share.^[^ ^3^ ^]^ The oxide material is usually mixed with carbon‐based conductive additive and binder and is coated on aluminum (Al) current collector. The electrolyte usually consists of lithium salts such as lithium hexafluorophosphate (LiPF_6_) and additives dissolved in carbonate‐based organic solvents. Last, the separator is commonly microporous polypropylene or polyethylene with or without ceramic coating. Several LIB cells are connected in parallel and/or series to form a module, and the modules are connected together, incorporated with battery management system (BMS) and thermal management system to form a battery pack.^[^ ^7^ ^]^ A typical LIB pack contains ≈25% by weight of casing and wiring, 20–25% anode material, 17–30% cathode material, 10–15% electrolyte, 5–15% copper foil, 5–10% aluminum foil, and 3–5% separator.^[^ ^8^, ^9^ ^]^

**Figure 1:**
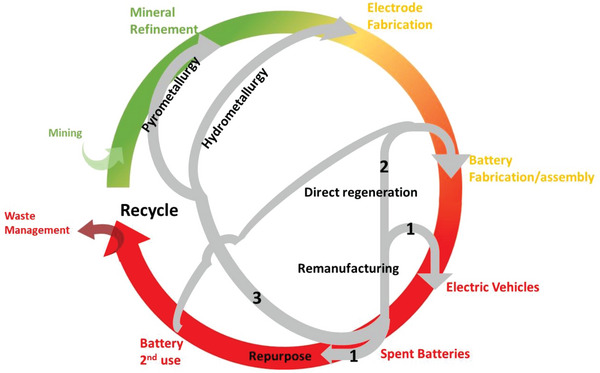
Circular life cycle of lithium ion‐batteries. The upstream, midstream, and downstream processes are represented in green, yellow, and red, respectively.

After retiring from EVs, a few choices are available for the spent LIBs depending on their state‐of‐health (SOH) and remaining useful life (RUL), as shown in **Figure** **1**. For the LIB packs that are still functioning with 70–80% of their initial capacity, they can be repurposed and reused in less demanding applications such as on‐grid or off‐grid energy storage systems (ESSs), following route 1 in Figure 1. If the packs do not meet the 80% capacity requirement due to some damage cells while the rest of the pack can still function well, the damaged cells could be replaced, and the battery pack can be reused in EV application after remanufacturing. This battery second use (B2U) is considered to be promising both from economic and environmental perspectives as the battery can be directly utilized after passing required performance and safety inspections without any complicated process or after minimal disassembling and remanufacturing.^[^ ^3^, ^10^ ^]^ It has been estimated that the B2U market could reach 26 GWh by 2025 and 1.01 TWh in 2063.^[^ ^11^ ^]^ The environmental advantage of B2U is emphasized by the fact that production of a new EV battery can emit up to 16 000 kgCO_2_ equivalent, which is nearly half of the whole EV manufacturing process.^[^ ^12^ ^]^ In the event that the battery packs do not meet the performance and safety requirements to be directly reused, they can be disassembled, undergo direct regeneration to repair the electrode materials and other components before returning to battery fabrication and assembling process (route 2). Finally, the heavily damaged end‐of‐life (EOL) battery packs can undergo recycling process (route 3) to recover valuable components such as lithium (Li), cobalt (Co), nickel (Ni), Cu, and Al.^[^ ^3^, ^13^ ^]^ This review comprehensively discusses these three routes for reuse, repair, and/or recycle of the spent LIBs from EVs focusing on technological availability, environmental impacts, and economical feasibility of the processes.

## 2 Second Use of Li‐Ion Batteries from Electric Vehicles

After being decommissioned from EVs, battery packs and/or modules are needed to be stabilized/discharged, transported, and evaluated before they can be reused in EV or other applications. The key steps in this process are to collect, inspect, evaluate, and sort the battery packs and modules. Because SOH of a spent LIB depends on various factors including electrodes/electrolyte compositions, driving patterns, operating temperatures, and charging procedures, it is therefore challenging to predict battery aging behavior without in‐depth characterization, which is a time‐consuming and costly process. In addition, sophisticated sensors and information systems are needed to track battery utilization, adding production cost to LIBs.^[^ ^14^ ^]^ Several tests involving physical, electrochemical, and spectroscopic techniques are critical to evaluate the battery's SOH and RUL. Ideally, the battery modules are tested without disassembling to the cell level due to the high cost and safety hazard of disassembling and reassembling processes.^[^ ^3^, ^10^ ^]^ However, disassembling might be necessary for the LIB packs containing some small percentages of cells that fail the required performance and safety standards and needed to be replaced.

In this section, we discuss evaluation method of spent LIB before sorting for B2U or recycling.^[^ ^14^ ^]^ In general, the spent LIB is evaluated in terms of safety, remaining capacity, internal resistance, self‐discharging rate, and cycle life. The first step is visual inspection to check whether there is any recognizable bulging, deformation, and/or leakage of electrolyte. If a LIB contains any visual damage, its possibility of being reused is immediately eliminated. Although this process is relatively easy and could be done quickly, it is labor‐intensive and has high risk of human error. Hence, advanced image processing algorithms are being developed to automate this step.^[^ ^15^ ^]^ After visual inspection, historical operation parameters of a LIB are evaluated. The criteria for this step are; 1) ≤5 times overcharged above 4.5 V or overdischarged below 2.0 V, 2) ≤5 times operating at a temperature between 50 and 80 °C, 3) <8 years of operation, and 4) <60% self‐discharge after storing at room temperature for 3 h. Next, basic performance parameters of a LIB are evaluated following; 1) cutoff discharge voltage is within the specified value, usually in the range of 1.9–3.0 V (2.5 V for LFP and 2.75 V for NMC batteries), 2) internal resistance is ≤1.5 times its initial value and ≤5 mΩ, 3) at room temperature, discharge capacities at 0.3 and 0.5 C are higher than 75% and 70% of its original value, respectively, 4) discharge capacities at 0.3 C are higher than 85% and 60% at −20 ± 5 and 50 ± 5 °C, respectively, and 5) charge retention at room temperature, −20 ± 5 and 50 ± 5 °C are above 80%, 70%, and 70%, respectively after storage for 28 days. Finally, internal microstructure of a LIB is inspected using non‐destructive computed tomography and the anode is analyzed by ^7^Li magnetic resonance imaging to observe whether there is any Li dendrite formation. The Li content on carbon anode must be ≤15 wt% to pass the safety criteria. Although the above criteria can effectively sort spent LIBs for B2U, standardized protocol and regulation for evaluation and sorting of LIBs are still lacking.^[^ ^1^, ^14^ ^]^ The development of such protocol should consider universality, reliability, processing time, and cost‐effectiveness. In addition, some key technologies relating to prediction and simulation of SOH and RUL of spent LIBs as well as non‐destructive safety testing processes still need to be further developed.^[^ ^3^, ^4^ ^]^

Economically, benefit of B2U depends on many factors, including the cost of the spent LIBs, logistics, storage, testing and sorting, the second use application, and maintenance.^[^ ^11^ ^]^ Several studies have shown that the cost of the spent LIBs and the labor cost dominate the overall cost of B2U value chain. As such, the estimated price for second life LIBs can range from 25 to $250 kWh^−1^ depending on the cost of spent batteries, wages, and other factors.^[^ ^3^, ^11^, ^16^ ^]^ However, this number is expected to be lower with lowering price of new batteries along with increase in EV utilization. To put into a perspective, the price of LIBs has decreased from nearly $1000 kWh^−1^ in 2010 to currently around $140 kWh^−1^ and is projected to be cheaper than $60 kWh^−1^ in 2030.^[^ ^2^ ^]^ Although there is an on‐going debate whether profitable B2U can be realized,^[^ ^11^ ^]^ some specific applications, such as demand charge and time‐of‐use services (DC&TOU) utility peaker plant replacement were considered to have high potential with sufficient market to allocate the spent LIBs.^[^ ^11^, ^16^ ^]^ Nonetheless, most B2U is currently still at a pilot scale or demonstration project, and the economic feasibility must be thoroughly evaluated before it can be deployed at an industrial level. Only some commercial products utilizing spent LIB are developed. For examples, Nissan and Eaton jointly developed xStorage, a 4.2‐kWh ESS using retired Nissan Leaf batteries that has been implemented at the Amsterdam arena. Also using retired Nissan Leaf batteries, Nissan partnered with Freewire Technologies to develop “Mobi,” a 48‐kWh mobile ESS.^[^ ^11^ ^]^

Moreover, another indirect benefit of B2U is the reduction of EV upfront costs, in which up to 25% reduction of battery upfront cost was estimated when B2U is taken in consideration. However, this factor is expected to be less significant over time as the cost of new batteries tend to decrease with the economies of scale.^[^ ^11^ ^]^ Overall, the concept of B2U is potentially profitable in some specific stationary energy storage applications with clear environmental benefits compared to other types of energy storage/production systems. However, there are some factors, including new LIB price reductions, absence of standardized testing procedure, and expensive repurposing and remanufacturing cost that are the deciding factors to determine the success or failure of this business model. Therefore, B2U needs strong political incentives and strict environmental policies to subsidize for these uncertainties and accelerate the adoption of B2U business model.^[^ ^16^ ^]^

## 3 Electrode Repair and Material Recovery

At the EOL of an EV battery pack, whether it happens after its first or second use, the electrode materials could be repaired by direct regeneration (route 2) or the valuable compositions could be recovered via recycling processes (route 3). For both routes, the EOL EV packs must undergo pretreatment process to safely access the electrode materials.

## 4 Pretreatments

Pretreatment process refers to deactivation, dismantling, and separation of an EV battery pack. The goals of deactivation step are to reduce the risk of electrical shock from the high voltage by discharging the battery through an external resister or submerging it in a salt solution, and to reduce the reactivity of the chemically active components in LIB by freezing in liquid nitrogen and/or operating in an inert atmosphere. Typically, NaCl solution is used to chemically discharge LIBs, but recent study has shown that FeSO_4_ is a more environmentally friendly choice.^[^ ^17^ ^]^ This process is a direct waste of residual energy stored in a battery and could take up to 5 h to reduce the voltage of LIB from 3.5 to 0.5 V, which is equivalent to electrically discharging at 0.2 C.^[^ ^17^ ^]^ Surprisingly, only a few companies such as Duesenfeld and TES‐AMM recovers this stored electricity via discharging the LIB into an ESS that powers the recycling facilities.^[^ ^18^, ^19^ ^]^ After discharged, a LIB pack can be directly shredded or manually dismantled into smaller components. While the former process is quick, cheap, and can be readily applied at an industrial scale, it increases the complexity of the material recovery process as all the components are mixed together. Some processes based on sieving, density, and wettability have been developed to increase the separation efficiency and overall yield.^[^ ^20^, ^21^ ^]^ On the other hand, the latter process allows for recovery of valuable component such as electrical wiring, sensors, and BMS. This process also allows for separation of electrode materials from the Al and Cu current collectors by solvent/alkaline dissolution coupled with ultrasonic‐assisted delamination. Moreover, washing the electrode materials with proper solvents after delamination to remove any additives and binders could improve the overall purity of the obtained materials.^[^ ^22^, ^23^ ^]^ Nonetheless, manual disassembling requires a skilled technician and specialized equipment as battery disassembly involves working with high voltage as well as flammable and toxic chemicals. This step significantly increases the cost of operation, especially in countries with relatively expensive labor cost. Although robotic and automated disassembling processes are being explored,^[^ ^24^ ^]^ their success in industrial scale adoption relies on standardized design of the battery packs and are typically done by EV manufacturers.

## 5 Direct Regeneration of Electrode Materials

Direct regeneration or direct recycling is referred to a recovery method to repair the crystal structure of an electrode by relithiation. Often times, it is done via solid‐state sintering, hydrothermal, electrochemical, and/or chemical processes. This process is more suited for spent LIBs which their active materials are not heavily damaged and is applicable to most electrode chemistries given that the electrode compositions are known. Typically, the electrode is removed from a battery by physical separation followed by mild thermal processing or solvent dissolution to remove the polymeric binder and detach the active material from the current collector. In the solid‐state sintering, the electrode materials are heating with a Li source, typically Li_2_CO_3_, Li_3_PO_4_, and LiOH at a temperature around 600–800 °C to replenish Li in the electrode structure.^[^ ^13^, ^25^ ^]^ Moreover, it has been shown that heat treatment can also convert the undesired spinel and rock salt phases of the spent NMC electrode back to its preferred layered structure.^[^ ^26^ ^]^ Direct regeneration has several advantages such as being a relatively easy and less complicated process, produce significantly less pollution compared to pyro‐ and hydro‐metallurgy, and the obtained materials have high electrochemical performance comparable or even better than the pristine material that can be directly used in the battery fabrication process (Figure 1). On the other hand, the disadvantages of this process include the need of information on the exact material chemistry of the electrodes and intensive sorting processes. In addition, economic feasibility of this process depends strongly on the price of Li source used in the relithiation process. Direct recycling is still at a research stage as it has not been proven to be profitable in industrial scale and its environmental benefits are positive only when applied to some specific type of batteries and cell designs.^[^ ^7^, ^27^ ^]^ Recent analysis reveals that direct regeneration of LFP batteries results in net increase in CO_2_ emission, citing that the iron used in LFP cathodes is more environmentally friendly to obtain by mining than via direct regeneration.^[^ ^27^ ^]^

## 6 Recycling of End‐Of‐life Li‐Ion Batteries

The idea of “urban mining” by recycling of LIBs to recover precious metals from the electrodes, current collectors, as well as used electrolyte is crucial to complete the circular economy loop of LIBs. Considering a typical NMC type LIB, there are 5–20% Co, 5–10% Ni, 10–15% Mn, 5–7% Li, 15% organic chemicals, and 7% plastic by weight.^[^ ^1^, ^22^ ^]^ Among these elements, Li, Co, and Ni have high economical value with the price of LIB‐grade LiOH, Co, and Ni metal of $72, $52, and $22 kg^−1^ (price as of September 2022, The London Metal Exchange), respectively, whereas organic components and plastic are of environmental concerns.^[^ ^28^ ^]^ Equally important, the prices of these metals are subjected to dynamic fluctuation due to geopolitical and economic situations, which can complicate the LIB value chain. For example, the price of Co has tripled from $25 to $82 kg^−1^ and reduced to around $52 kg^−1^ over the course of 2 years.^[^ ^28^ ^]^ Stimulated by this motivation, there are currently more than 21 established and 11 planned LIB recycling facilities with a combined capacity of more than 300 000 tons located in east Asia, Europe, and North America.^[^ ^5^ ^]^

**Figure 2:**
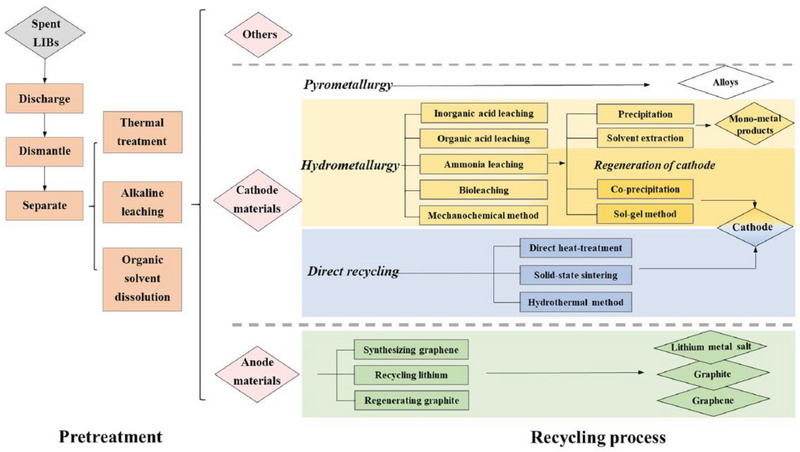
Flow chart of recycling processes of lithium‐ion batteries (LIBs). Reproduced with permission.^[^ ^13^ ^]^ Copyright 2021, Elsevier.

Due to the complex structure with multiple components of a LIB pack, its recycling process consists of several steps that are different depending mostly on available technology and type of LIB being recycled, as shown in **Figure** **2**. Generally, the EOL LIBs undergoes pyro‐ or hydro‐metallurgy or a combination of both methods. Additionally, there are currently active research activities surrounding electrochemical recycling of the electrode materials. Here, we discuss the overview of each process, its environmental impact, economical assessment, and some examples being used in LIB recycling industries.

**Figure 3:**
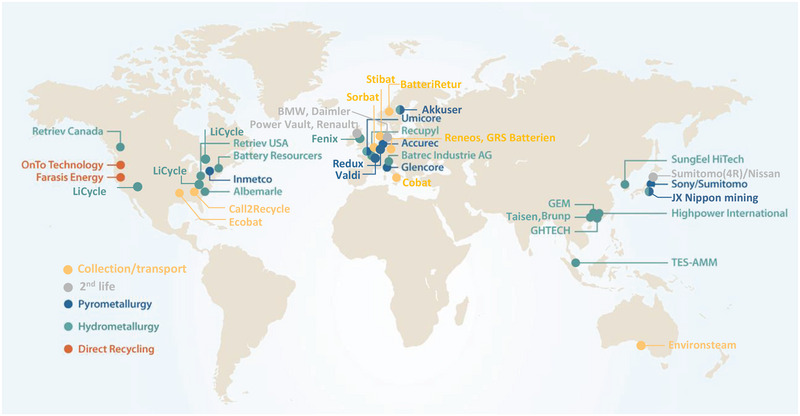
A map of recycling facilities including companies providing collection and transport services as well as battery second use projects. Adapted with permission.^[^ ^30^ ^]^ Copyright 2019, Cell Press.

Pyrometallurgy refers to a process of heat treatment to convert metal oxides to metals or metal alloys under a reductive atmosphere. It can be used to recover Co, Ni, Fe, and Cu metals, while Li, Al, and Si fall into the slag that need to be further leached and extracted with additional cost and energy. The output of the process is elemental component that can be further refined before re‐entering the LIB life cycle (Figure 1). Pyrometallurgy is relatively easy, requires less pretreatment process, and is applicable to recovery of both the anode and the cathode materials. Additionally, it does not involve determining battery's design, compositions, and state of charge prior to recycling. However, it consumes large amount of energy to maintain the high temperature of the smelting furnace (≈1000 °C) and emits hazardous and greenhouse gases. Moreover, plastic components (casing and separator) and organic electrolyte, which correspond to nearly half of the total weight of a battery, are not recovered by this process, except in some special process such as that developed by Accurec Recycling GmbH (Accurec).^[^ ^29^, ^30^ ^]^ Economically, a model has been proposed for recycling of 10 tons/day of spent cylindrical LMO batteries, and a daily profit of ≈$2100 could be obtained using in situ reduction pyrolysis.^[^ ^31^ ^]^ Due to high capital expenditures (CAPEX) and operating expenses (OPEX), this process may have poor economic feasibility for LIBs with low price cathode materials such as LFP or those with low Co content.^[^ ^30^ ^]^ Pyrometallurgy has high technical maturity and has been adopted by several companies including Umicore, Glencore, and Sumitomo/Sony, as shown in **Figure** **3**.^[^ ^5^, ^30^ ^]^ Despite its high maturity, constant developments of the process are being reported, mainly to reduce energy consumption and to increase Li and other metals recovery from slag.^[^ ^30^ ^]^

Because the products of pyrometallurgy are metal oxides, intermetallic alloys, and soluble Li salts, which cannot readily be used for electrode fabrication, the resulting products from pyrometallurgy usually undergo further leaching by hydrometallurgy processes to obtain lithium carbonate or lithium hydroxide monohydrate that are then returned to the LIB value chain. For example, the Umicore battery recycling process is applicable for both LIB and nickel metal hydride batteries without mechanical pretreatment, producing alloys of Co, Ni, and Cu that are further processed to precursors for new cathode materials by hydrometallurgical process obtaining over 95% yield.^[^ ^29^, ^32^ ^]^ More importantly, the process can recover most of the lithium, solving a key constraint of a typical pyrometallurgy process. Its recycling plant in Hoboken, Belgium has a capacity of 7000 tons of LIBs, equivalent to 35 000 EV batteries per year.^[^ ^32^ ^]^ In addition to Umicore, a German recycling company based in Krefeld, Accurec also uses a combination of mechanical, pyro‐, and hydro‐metallurgical processes to recover Co–Ni–Mn alloy as well as Li_2_CO_3_. The Accurec process can also recover electrolytes and other volatile solvents from the spent LIBs, as will be discussed further in the relevant section.^[^ ^29^ ^]^

Hydrometallurgy involves leaching of electrode components into solution followed by separation and purification. The chemistry of the process varies depending on the types of LIBs, compositions of the electrodes, and the leaching agents used. It can be categorized into three types based on the leaching media, that is, acid leaching, ammonia leaching, and bioleaching. For acid leaching, strong inorganic acids such as H_2_SO_4_, HCl, and HNO_3_ combined with reducing agent such as H_2_O_2_ are commonly used due to their high leaching efficiency with mild conditions and low cost.^[^ ^33^ ^]^ This process, however, generates toxic gases, release acidified waste water, and requires large amount of water to neutralize the leachate.^[^ ^22^ ^]^ To address the environmental concerns of the process, organic acid such as citric, lactic, oxalic, and ascorbic acids can be used instead. Benefiting not only from the acidity of the organic acids but also their chelating and reducing properties to stabilize and reduce the leached metals, organic acid leaching can reach similar efficiencies to inorganic acid.^[^ ^3^ ^]^ As such, organic acid leaching can reduce the steps needed to separate leached metals by combining leaching and precipitation into one step, which has both environmental and economic benefits.^[^ ^34^ ^]^ Similarly, ammonia leaching employs selective chelating property of ammonia with Ni^2+^, Co^2+^, and Mn^2+^ at different pH values.^[^ ^13^ ^]^ Importantly, the ammonia from used leaching agent can be recovered by ammonia distillation, providing additional environmental and economic benefits of the process.^[^ ^35^ ^]^ Last, chemolithotropic and acidophilic bacteria such as *Acidithiobacillus ferrooxidans* and *Aspergillus niger* are used to dissolve metals from the spent electrode in bioleaching process. Although this process benefits from low energy consumption and environmental perspective, the slow leaching kinetics and high potential of contamination limits its practical use in industries.

After leaching, metals or metal compounds are separated from the leachate via filtration, chemical precipitation, solvent extraction, ion exchange, and/or electrolysis.^[^ ^13^ ^]^ Typically, carbon impurities are removed by filtration as they are not soluble in the leachate aqueous medium. Then the metal impurities such as Al and Cu from the current collectors are removed by lowering the pH of the leachate. To precipitate the transition metals, NaOH, oxalic acid (H_2_C_2_O_4_), Na_2_S, and Na_2_CO_3_ are commonly used as precipitants. Alternatively, the metal ions are separated using stepwise solvent extraction based on different solubility in aqueous and organic phases. Common solvents for precipitation of Co^2+^, Ni^2+^, and Mn^2+^ are PC‐88A, Cyanex 272, and D2EHPA.^[^ ^13^ ^]^ This process, however, suffers from high cost of the solvents and corrosion of equipment. Finally, Li^+^ is precipitated out using Na_2_CO_3_, or H_3_PO_4_, or Na_3_PO_4_. Throughout the purification processes, the precipitant concentration, pH, temperature, and time need to be optimized and strictly controlled to achieve the highest yield and purity of the recovered metals. Hydrometallurgy holds several advantages including its ability to recover almost all the components of the spent LIBs with high purity and being less energy intensive compared to pyrometallurgy. However, it employs highly toxic chemicals and large amount of water are used in the process. In addition, it produces significant amount of landfill waste containing metals and salts. Because of its tempting advantages, hydrometallurgy is being deployed by several companies and startups, including Retriev, Recupyl, Battery Resoucers, and Li‐Cycle (Figure 3).^[^ ^5^, ^30^ ^]^ For example, the process developed by Retriev begins with manually disassembling large LIB packs before crushing them while submerging in a brine solution to reduce the battery activity and prevent gas emission. After this process, three types of materials are produced which are plastics, metal solid, and metal‐enriched solution. The light metal and plastics are separated by a shaking table, while the metal solid part is filtered to obtain a carbon‐ and metal oxide‐rich cake that are sold for further Co and Ni extraction. The Li in the metal‐rich solution is precipitated in the form of Li_2_CO_3_ using Na_2_CO_3_.^[^ ^29^, ^30^ ^]^

**Figure 4:**
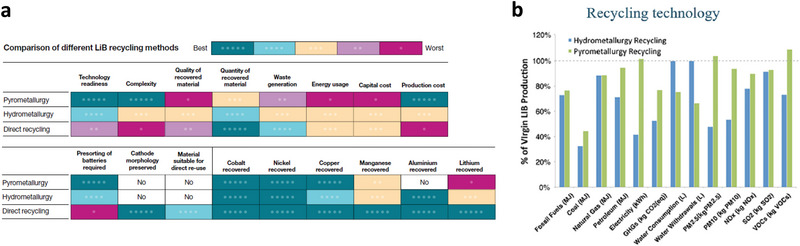
a) Comparison of LIB recycling methods. Reproduced with permission.^[^ ^33^ ^]^ Copyright 2019, Springer Nature. b) Environmental impact of hydrometallurgy and pyrometallurgy compared to production of LIBs using virgin materials. Reproduced with permission.^[^ ^3^ ^]^ Copyright 2020, ACS Publications.

Comparisons of the overall process and environmental impacts between pyro‐ and hydro‐metallurgy are shown in **Figures** **4**a,b, respectively.^[^ ^3^, ^33^ ^]^ Compared to LIBs produced from newly mined materials, hydrometallurgy can reduce to overall environmental impact in the area of primary energy consumption and greenhouse gas emission by roughly 40%, whereas the number is ≈20% for pyrometallurgy.^[^ ^3^ ^]^ In addition, it has been shown that using Ni and Co recovered from a recycling process could save about 51% of the natural resource needed in a battery production chain compared to the virgin resource supply.^[^ ^36^ ^]^ Although both the pyro‐ and hydro‐metallurgy have been commercialized, their business model depends strongly on the type and compositions of the LIBs being recycled as the amount of valuable Co tend to be lower for modern LIBs, and the price of new LIBs become much cheaper with increasing large scale production. For instance, rough estimation of profit gains from recycling of 1 ton of LMO, LFP, LCO, and NMC batteries using either pyro‐ or hydro‐metallurgy are $432, $196, $28 016, and $5013, respectively.^[^ ^3^ ^]^ Nonetheless, these number can change significantly depending on equipment, utility, labor costs, and market prices of the obtained metals.^[^ ^3^ ^]^ Therefore, choosing a suitable process for recycling of a certain type of LIB is critical to yield reasonable profits. Additionally, the supply chain management for collection, transportation, and storage of spent LIBs still needs to be further developed, taking into account material volumes, collection rates, and cost, as battery recycling facilities require high CAPEX and OPEX, so high volume of the EOL batteries is needed for the process to become economically feasible.^[^ ^30^, ^37^ ^]^

Electrochemical process has gained increasing attention as an alternative method to recover precious metals from spent LIBs due to its fast‐processing time, high recovery rate, and versatility of the processing parameters.^[^ ^38^, ^39^ ^]^ In one study, Li, Ni, Co, and Mn were recovered from NMC 111 cathode with efficiencies exceeding 99.5% within 30 min via cathode reduction in malic acid. The process could also be adapted to extract metals from other cathode chemistries, such as LMO and other compositions of NMC.^[^ ^38^ ^]^ The electrochemical lithium extraction has also been proposed by a Singapore‐based startup, NEU Battery Materials that claimed to have profitable process for recovery of Li from LFP cathodes.^[^ ^39^ ^]^ Although electrochemical processes hold promises in metal recovery from spent LIBs, electricity consumption adds another layer of complexity in economic feasibility of the overall processes. The cost of electricity becomes one of the determining factors for the profitability of the process, and the source of electricity influences its environmental footprint. Electricity from alternative energy such as solar and wind should be efficiently utilized in the electrochemical metal extraction process.

## 7 Anodes

Currently, anode is not the main focus of LIB recycling due to low value of the anode materials (mostly graphite) and Cu foil current collector. However, there has been an increasing interest in recovery of graphite as well as other anode compositions because of their high abundance in a spent LIB. For instance, an average EV LIB contains ≈25 wt% or roughly 25–70 kg of graphite that either ends up in landfill or incinerated after use.^[^ ^9^, ^40^, ^41^ ^]^ Moreover, ≈70% of the global graphite production for EVs is controlled by China, which contributes to the risk of supply instability as a result of increasingly concerning geopolitical conflicts.^[^ ^30^ ^]^ Furthermore, graphite purification process requires usage of strong acids and hence is not environmentally friendly.^[^ ^41^ ^]^ Recovered graphite can be reused as an active material in LIB anode after processing or converted to high value materials such as graphene and reduced graphene oxide that can be utilized in several applications including sensors, catalysts, and supercapacitors.^[^ ^9^, ^40^ ^]^ Additionally, it has been shown that exfoliation of graphite from spent LIBs can be done with milder conditions compared to natural graphite due to the expanded interlayer spacing from Li‐intercalation and the presence of structural defects.^[^ ^42^ ^]^ In addition to graphite, ≈5–7 wt% of anode consists of Li, which could further accelerate the recycling of anode given the quickly increasing price of battery‐grade Li_2_CO_3_ and LiOH.^[^ ^41^ ^]^

To recover graphite, hydrometallurgy or direct recycling process is preferred over pyrometallurgy as graphite along with other organic components of a LIB are incinerated as fuel in the latter process.^[^ ^9^ ^]^ Generally, graphite is recovered via washing, thermal treatment, and a hybrid process using acid leaching and thermal treatment processes.^[^ ^40^ ^]^ Ideally, the anode sheet should be separated from the rest of the LIB components and graphite particles are delaminated from the Cu current collector by crushing, solvent washing, and mild thermal treatment owing to weak binding between graphite and Cu.^[^ ^43^ ^]^ However, the anode is usually crushed together with the cathode and other component, generating the black mass, in most processes. In this case, graphite needs to be separated after acid leaching and filtration, as described in the hydrometallurgy section. To recover graphite by the washing process, the obtained graphite powder after separation can be washed with a solvent such as dimethyl carbonate (DMC) followed by *N*‐methylpyrrolidone (NMP) to remove the lithium salt and PVDF binder. For the thermal treatment process, the anode sheet is cut into thin slices and heated at 1400 °C. This process evaporates the contaminated solvents, decomposes binder, and smelts Cu current collector into copper spheres that can be sieved out of the remaining graphite powder. Thermal treatment at higher temperature (2000–3000 °C) can also be used to increase graphitization degree of the obtained graphite powder.^[^ ^44^ ^]^ Finally, acid leaching is used instead of solvent washing followed by thermal treatment in the hybrid processes. In addition to the aforementioned methods, electrolysis, supercritical extraction, and microwave treatment are being explored to find a more efficient and economical method for graphite recovery.^[^ ^40^ ^]^

Industrial demonstration of graphite recovery can be seen in the process developed by the OnTo technology LLC., where graphite is recovered under supercritical condition followed by thermal treatment and the obtained graphite with the same size and morphology as the commercial graphite. The process, however, needs to be further developed for large scale operation in terms of environmental impact and cost.^[^ ^9^ ^]^

## 8 Electrolytes

Electrolyte in spent LIBs is present in both a liquid and a solid form that is immobilized on the electrode as solid electrolyte interphase. It contains significant amount of Li in the form of toxic Li salts and flammable organic solvents. The extracted electrolytes usually contain a mix of original solvents and additives as well as their decomposed products,^[^ ^45^ ^]^ rendering reuse and/or recycling them challenging and thus expensive. In addition, the Li salt in the electrolyte is usually recovered in trace quantity.^[^ ^3^ ^]^ Therefore, separation of electrolytes from spent LIBs has more environmental benefits over economic benefits. In fact, electrolyte as well as other less valuable parts of the LIBs (casing, wiring, and circuits) are being recycled only to meet the new regulation recently announced in United States, the European Union (EU), and China.^[^ ^46^ ^]^

Different extraction methods have been proposed to extract electrolyte from spent LIBs, such as thermal evaporation, solvent extraction, electrolyte extraction by subcritical CO_2_ and acetonitrile, and electrolyte extraction with supercritical CO_2_.^[^ ^3^, ^45^ ^]^ The results suggest that extraction with subcritical and supercritical CO_2_ is among the most promising methods, as they do not introduce more impurities to the extracted products, in contrast to solvent extraction method. Moreover, it has been demonstrated that the yield of recovered electrolytes depends on the types of extraction methods, where linear carbonates such as DMC and EMC are more efficiently recovered with liquid CO_2_ while cyclic carbonate such as EC is more efficiently extracted with supercritical CO_2_.^[^ ^3^ ^]^ On a safety note, LIB electrolyte generates highly toxic hydrofluoric acid when exposed to water. Therefore, the extracted electrolyte must be purified by anion exchange resin, so the amount of hydrofluoric acid is below the toxic level.

Industrially, Accurec developed a vacuum pyrolysis recycling technology that can collect electrolyte and volatile solvents using vacuum heat treatment below 250 °C followed by condensation, while OnTo Technology LLC uses supercritical CO_2_ to extract electrolytes, which results in less impurities compared to solvent extraction.^[^ ^29^ ^]^ In addition to liquid electrolytes, there are also some recent efforts in recycling of the garnet‐type solid‐state electrolyte, lithium lanthanum zirconium oxide solid‐state electrolyte by combination of mechanical and hydrometallurgical methods, but this relatively new concept still needs to be proven at pilot and industrial scale.^[^ ^47^, ^48^ ^]^

## 9 Conclusion and Perspectives

Reuse and recycle of spent and EOL LIBs are presently needed and the urgency of having sustainable, profitable, and environmentally friendly processes is growing rapidly. However, most of the business models proposed for reuse and recycle of LIBs available to date have high uncertainty in terms of profitability, partly due to the complexity of the cost structure and value chains of the new and used LIBs. B2U is the most suitable route for off‐spec LIBs and the battery packs with substantial residual capacity after retired from EV application. Although this process offers the most direct route of “extracting” the residual value of a battery that could potentially reduce the upfront costs of EVs, it is not widely adopted because of questionable profitability of the process that is partly due to lack of standardized testing and sorting, high cost, and low volume of spent LIBs that are suitable for B2U, in addition to high cost of transportation as well as handling. Direct regeneration of electrode materials by relithiation and thermal treatment offers a way to directly recycle cathode materials and return the materials back to battery fabrication process with relatively low‐cost and low environmental footprint. For both processes, having information regarding electrode chemistries and battery operation histories is essential and can significantly improve the reuse/recycling processes. This requires the battery manufacturers to design and implement efficient standardized battery labeling systems as well as employ BMS systems that can realize real‐time monitoring of the battery SOH and transmit the data to a server that is accessible to recyclers. This option is being explored by a few companies including TÜV SÜD. The idea of “battery passport,” where the manufacturers must provide durability and performance data for their batteries and are responsible for the provenance of battery materials is being considered in the EU. It also demands the manufacturer to disclose the component of the batteries and where the materials come from as well as their environmental impacts. Quite surprisingly and concerning, many countries do not yet have regulation framework in place for recycling of LIBs.^[^ ^46^ ^]^ As importantly, it is important to urge battery manufacturers to design ready‐to‐recycle batteries, which could be done through implementing environmental policies and/or offering tax incentives.

For the EOL battery, electrode materials as well as other valuable parts can be recovered via pyrometallurgy and/or hydrometallurgy. On the one hand, pyrometallurgy is technologically more mature with relatively simpler process that does not require intensive sorting and pretreatment of the spent LIBs. However, it can only recover some metals such as Co, Ni, and Fe leaving valuable Li in the slag that needs to be further recovered. In addition, the process involves incineration of plastics and toxic electrolytes, raising concerns on its environmental impacts. On the other hand, hydrometallurgy allows recovery of almost every battery composition with relatively high purity while being less energy intensive compared to pyrometallurgy, but the process uses highly corrosive chemicals and produces large amount of landfill wastes as well as acidified wastewater. Novel approaches and process optimization are being developed to reduce costs, process time, environmental impacts, and eventually to make the processes more economically feasible for widespread adoption. Finally, recycling of LIBs has excellent examples of successful lead‐acid battery recycling to follow, where their recycling rates reach almost 100% both in the United States and Europe via a value‐driven model. Despite much more complicated cathode chemistries, complex cell and pack structures, and potentially harmful components, successful recycling of EV LIBs could soon be realized given that sustainable recycling technology and profitable business models are developed.

## Conflict of Interest

The authors declare no conflict of interest.
